# NeoBOMB1, a GRPR-Antagonist for Breast Cancer Theragnostics: First Results of a Preclinical Study with [^67^Ga]NeoBOMB1 in T-47D Cells and Tumor-Bearing Mice

**DOI:** 10.3390/molecules22111950

**Published:** 2017-11-11

**Authors:** Aikaterini Kaloudi, Emmanouil Lymperis, Athina Giarika, Simone Dalm, Francesca Orlandi, Donato Barbato, Mattia Tedesco, Theodosia Maina, Marion de Jong, Berthold A. Nock

**Affiliations:** 1 Molecular Radiopharmacy, INRASTES/NCSR “Demokritos”, 15310 Athens, Greece; katerinakaloudi@yahoo.gr (A.K.); mlymperis@hotmail.com (E.L.); athina.giarika@gmail.com (A.G.); maina_thea@hotmail.com (T.M.); 2 Department of Radiology, Erasmus MC, 3015 CN Rotterdam, The Netherlands; s.dalm@erasmusmc.nl (S.D.); m.hendriks-dejong@erasmusmc.nl (M.d.J.); 3 Advanced Accelerator Applications, 10010 Colleretto Giacosa TO, Italy; francesca.orlandi@adacap.com (F.O.); donato.barbato@adacap.com (D.B.); mattia.tedesco@adacap.com (M.T.)

**Keywords:** GRPR-antagonist, theragnostics, targeted tumor imaging, PET-imaging, breast cancer

## Abstract

Background: The GRPR-antagonist-based radioligands [^67/68^Ga/^111^In/^177^Lu]NeoBOMB1 have shown excellent theragnostic profiles in preclinical prostate cancer models, while [^68^Ga]NeoBOMB1 effectively visualized prostate cancer lesions in patients. We were further interested to explore the theragnostic potential of NeoBOMB1 in GRPR-positive mammary carcinoma, by first studying [^67^Ga]NeoBOMB1 in breast cancer models; Methods: We investigated the profile of [^67^Ga]NeoBOMB1, a [^68^Ga]NeoBOMB1 surrogate, in GRPR-expressing T-47D cells and animal models; *Results*: NeoBOMB1 (IC_50_s of 2.2 ± 0.2 nM) and [^nat^Ga]NeoBOMB1 (IC_50_s of 2.5 ± 0.2 nM) exhibited high affinity for the GRPR. At 37 °C [^67^Ga]NeoBOMB1 strongly bound to the T-47D cell-membrane (45.8 ± 0.4% at 2 h), internalizing poorly, as was expected for a radioantagonist. [^67^Ga]NeoBOMB1 was detected >90% intact in peripheral mouse blood at 30 min pi. In mice bearing T-47D xenografts, [^67^Ga]NeoBOMB1 specifically localized in the tumor (8.68 ± 2.9% ID/g vs. 0.6 ± 0.1% ID/g during GRPR-blockade at 4 h pi). The unfavorably high pancreatic uptake could be considerably reduced (206.29 ± 17.35% ID/g to 42.46 ± 1.31% ID/g at 4 h pi) by increasing the NeoBOMB1 dose from 10 pmol to 200 pmol, whereas tumor uptake remained unaffected. Notably, tumor values did not decline from 1 to 24 h pi; Conclusions: [^67^Ga]NeoBOMB1 can successfully target GRPR-positive breast cancer in animals with excellent prospects for clinical translation.

## 1. Introduction

Molecular imaging and radionuclide therapy of cancer can be achieved by means of radiolabeled peptide analogs, radiopeptides, directed at receptor targets overexpressed on tumor cells [1,2]. After injection of a peptide radiolabeled with a gamma (^99m^Tc, ^111^In) or a positron emitting radionuclide (^68^Ga, ^64^Cu) in patients, the radiolabel accumulates to pathological sites releasing a diagnostic signal. The latter allows reliable mapping of the disease with the aid of single photon emission computed tomography (SPECT) or photon emission tomography (PET), respectively. In the case of particle emitting radionuclides (^177^Lu, ^90^Y), radiotoxic loads are delivered to tumors causing cell apoptosis and death. By adopting an integrated “theragnostic” approach, a “patient-tailored”, more efficacious management of cancer becomes feasible [3,4]. In this concept, the diagnostic radiopeptide will (i) reveal sites of the disease; (ii) select patients that may benefit from targeted therapies; (iii) contribute in dosimetric calculations; and (iv) assist in therapy planning, whereas the therapeutic radiopeptide counterpart will carry out the actual therapy. Then, the diagnostic radiopeptide will be re-applied for assessment of therapeutic responses and disease follow-up. Thus far the theragnostic approach has been successfully applied in the treatment of neuroendocrine tumors with radiolabeled octreotide analogs and is rapidly expanding toward other classes of human tumors.

In this respect, the gastrin-releasing peptide receptor (GRPR) has attracted considerable attention as a promising biomolecular target in nuclear oncology, due to its high-density expression in frequently occurring human cancers (prostate and breast cancer) [5,6,7,8,9,10,11,12]. Diagnosis and staging of prostate and breast cancer essentially rely on biopsies that are often inconclusive. On the other hand, most conventional imaging modalities, such as computed tomography (CT) or magnetic resonance imaging (MRI), lack sensitivity and/or specificity. Hence, more accurate and non-invasive diagnostic tools are needed; for example, suitable nuclear medicine probes targeted at the GRPR. Most anti-GRPR radiopeptides have been hitherto developed for treatment of prostate cancer [13]. However, their value in breast cancer theragnostics may be equally high, and is currently being explored. Notably, high GRPR expression levels were documented in >60% of patient biopsy specimens of invasive breast carcinomas by GRPR-autoradiography, whereas all metastases originating from GRPR-positive primaries preserved high GRPR expression status [11]. These results were later confirmed, and were related to biochemical factors such as estrogen and/or progesterone receptor expression [14,15]. A very recent study investigating more than 1400 primary tumors from breast cancer patients emphasized the clinical relevance of GRPR as a target for breast cancer, especially in the case of estrogen receptor-positive tumors [16]. Accordingly, anti-GRPR radiopeptides for breast cancer theragnostics may soon become of high clinical impact.

For radiolabeling with theragnostic radionuclide pairs, natural GRPR-ligands, like the amphibian tetradecapeptide bombesin (BBN) or the human 27mer GRP and their C-terminal fragments, have served as motifs for the development of suitable peptide-analogs. In principle, peptide-conjugates are designed to accommodate a radiometal binding domain (or chelator) coupled, directly or via a linker, to the *N*-terminus of the peptide chain via an amide bond [13]. Soon afterwards new peptide motifs with receptor-antagonist profiles were adopted in anti-GRPR radiopeptide design, while a shift of paradigm from GRPR-radioagonists to GRPR-radioantagonists occurred in the field [17,18]. Radioantagonists possess higher inherent biosafety, because they do not activate the GRPR after administration to patients and hence, unlike agonists, they do not elicit adverse effects. In addition, GRPR-radioantagonists were most often able to achieve higher accumulation in GRPR-positive lesions and to clear faster from background tissues compared to radioagonists, displaying an overall more attractive pharmacokinetic profile in animal models and in patients.

**Figure 1:**
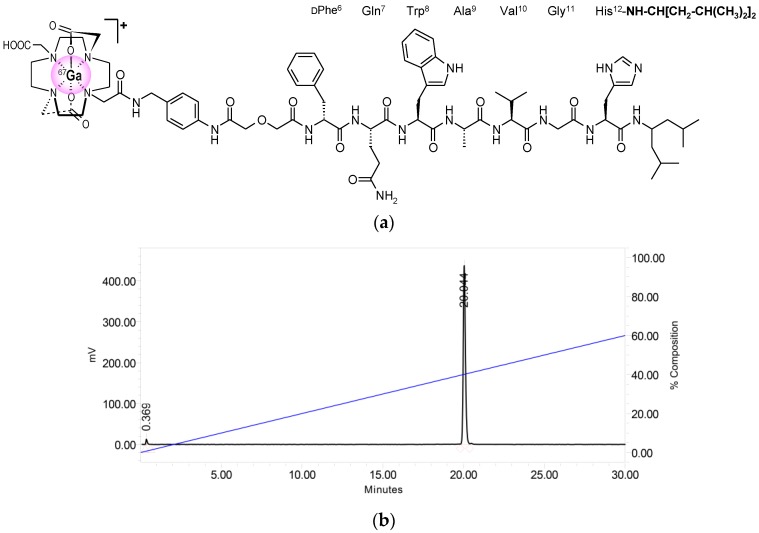
Radiolabeling of NeoBOMB1 with ^67^Ga: (**a**) Chemical structure of [^67^Ga]NeoBOMB1 radioligand; (**b**) Radiochromatogram of HPLC analysis of [^67^Ga]NeoBOMB1 labeling reaction mixture, showing a quantitative formation of high purity radioligand eluting at *t*_R_ = 20.0 min.

As a part of our search for theragnostic anti-GRPR radiopeptides for clinical use, we recently introduced NeoBOMB1 [19,20], based on the highly potent and metabolically stable GRPR-antagonist Ac-His-Trp-Ala-Val-Gly-His-NH-CH[CH_2_-CH(CH_3_)_2_]_2_ [21]. After replacement of His^1^ by Gln and elongation of the hexapeptide chain with DPhe, the universal chelator DOTA (1,4,7,10-tetraazacyclododecane-1,4,7,10-tetraacetic acid) was coupled to the *N*-terminal DPhe-amine via a *p*-aminomethylaniline-diglycolic acid linker (Figure 1a), thus allowing for labeling with ^111^In (for SPECT), ^68^Ga (for PET) and ^177^Lu (for radionuclide therapy). The resulting [^67/68^Ga/^111^In/^177^Lu]NeoBOMB1 radioligands showed excellent theragnostic profile during preclinical evaluation in prostate cancer models. Most importantly, [^68^Ga]NeoBOMB1 successfully visualized prostate cancer lesions in patients with PET/CT [19].

In the present study, we were interested in further exploring the diagnostic value of [^68^Ga]NeoBOMB1 in breast cancer with PET imaging. For practical reasons we decided to use the chemically identical [^67^Ga]NeoBOMB1 surrogate in our preclinical study, due to the convenient half-life of ^67^Ga (t_1/2_ = 3.3 d) compared to ^68^Ga (t_1/2_ = 68 min) [19]. Our in vitro and in vivo models were based on the human GRPR-positive T-47D cell line, concurrently expressing the estrogen receptor as well [14,22,23]. The results of the present study confirmed the ability of [^67^Ga]NeoBOMB1 to efficiently target experimental breast tumors in animals and revealed the excellent prospects of [^68^Ga]NeoBOMB1 for clinical translation in breast cancer patients.

## 2. Results

### 2.1. Peptides and Radioligands

NeoBOMB1 (Figure 1a) was labeled with ^67^Ga at specific activities of 3.7–7.4 MBq ^67^Ga/nmol NeoBOMB1 following a published method [19]. Quality control by radioanalytical HPLC revealed the formation of [^67^Ga]NeoBOMB1 in radiochemical labeling yield >98% and a radiochemical purity >99%; a representative radiochromatogram of [^67^Ga]NeoBOMB1 is shown in Figure 1b.

### 2.2. In Vitro Assays in T-47D Cells

#### 2.2.1. Affinity of NeoBOMB1 and [^nat^Ga]NeoBOMB1 for the GRPR

**Figure 2:**
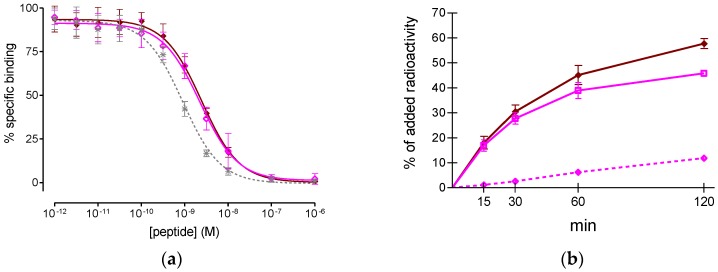
(**a**) [^125^I-Tyr^4^]BBN displacement curves from GRPR-sites on T-47D cells after 3 h incubation at 4 °C by ◆ [^nat^Ga]NeoBOMB1 (IC_50_ 2.5 ± 0.2 nM, *n* = 3), ◇ NeoBOMB1 (IC_50_ 2.2 ± 0.2 nM, *n* = 3) and ✴ [Tyr^4^]BBN (IC_50_ 1.33 ± 0.09 nM, *n* = 5); (**b**) Curves of time-dependent association of [^67^Ga]NeoBOMB1 to T-47D cells at 37 °C. Results represent average specific cell binding ± sd (◆ MB+I: ☐ membrane bound + ◇ internalized) vs. total added activity for each time point (*n* = 3, in triplicate); non-specific values were retrieved in the presence of 1 μM NeoBOMB1 and were subtracted from totals; the study was conducted with T-47D cells at 80–85% confluency.

As shown in Figure 2a, both non-metalated NeoBOMB1 and [^nat^Ga]NeoBOMB1 were able to displace [^125^I-Tyr^4^]BBN from GRPR-sites on T-47D cells in a monophasic and dose-dependent manner. The respective half-maximal inhibitory concentration (IC_50_) values were found indistinguishable (2.2 ± 0.2 nM and 2.5 ± 0.2 nM) and comparable to the [Tyr^4^]BBN reference (IC_50_ = 1.33 ± 0.09 nM). Thus, incorporation of Ga by the DOTA chelator in NeoBOMB1 did not negatively affect receptor affinity, confirming previous observations from studies in prostate adenocarcinoma PC-3 cell membranes [19].

#### 2.2.2. Time-Dependent Internalization of [^67^Ga]NeoBOMB1 in T-47D Cells

At 37 °C, [^67^Ga]NeoBOMB1 strongly and specifically bound to the cell-membrane of T-47D cells at all time points tested, reaching 45.8 ± 0.4% at 2 h, while only a small portion of radioactivity was internalized into cells (e.g., up to 12% at 2 h), which is consistent with a radioantagonist profile (Figure 2b).

### 2.3. In Vivo Evaluation of [^67^Ga]NeoBOMB1

#### 2.3.1. Stability of [^67^Ga]NeoBOMB1 in Healthy Mice

**Figure 3:**
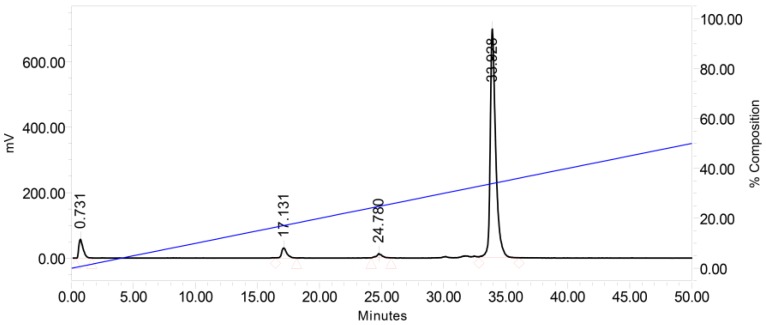
Radiochromatogram of HPLC analysis of mouse blood sample collected 30 min pi of [^67^Ga]NeoBOMB1, showing the presence of 90% intact [^67^Ga]NeoBOMB1 in peripheral mouse blood at *t*_R_ = 33.9 min, as determined by co-injection of a [^67^Ga]NeoBOMB1 sample in the HPLC.

Analysis of mouse blood samples collected at 5 min and 30 min pi showed that [^67^Ga]NeoBOMB1 remained 98% and 90% intact, respectively, in mouse circulation, displaying high metabolic stability. A representative radiochromatogram of a 30 min pi blood sample is shown in Figure 3.

#### 2.3.2. Biodistribution of [^67^Ga]NeoBOMB1 in Mice Bearing Human T-47D Xenografts

The biodistribution of [^67^Ga]NeoBOMB1 was studied in severe combined immune deficiency (SCID) mice bearing GRPR-positive human breast cancer T-47D xenografts. Development of well-palpable subcutaneous T-47D tumors in SCID mice was quite challenging, necessitating treatment of mice with estrogens for rather prolonged periods of time (approximately 8 weeks) [14]. The effect of injected peptide mass on biodistribution was first compared in two groups of mice at 4 h pi after injection of a 100 µL bolus [^67^Ga]NeoBOMB1 via the tail vein. Thus, the first group of animals received a total 10 pmol (at a specific activity of 3.7 MBq/nmol) and the second group a 200 pmol peptide (at a specific activity of 0.185 MBq/nmol) together with the radioligand; an additional third animal group received a high excess NeoBOMB1 (40 nmol) to assess GRPR-specificity of uptake. Results, as listed in Table 1, are expressed as percent injected dose per gram tissue (% ID/g) and represent mean values ± sd (*n* = 4). High uptake of radioactivity was seen in the mouse pancreas and the experimental tumor. This can be assigned to a GPRR-specific process, given that during in vivo GRPR blockade at excess 40 nmol peptide dose uptake was banned in both pancreas (1.17 ± 0.11% ID/g; *p* < 0.001) and tumor (0.64 ± 0.10% ID/g; *p* < 0.001). Notably, at the 200 pmol peptide dose, the uptake in the GRPR-rich mouse pancreas was significantly reduced (from 206.29 ± 17.35% ID/g to 42.46 ± 1.31% ID/g; *p* < 0.001), whereas tumor levels remained unaffected.

In view of the above, we further studied the biodistribution of [^67^Ga]NeoBOMB1 at 1 h, 4 h and 24 h pi in the same animal model at the 200 pmol peptide dose. Results are listed in Table 2 as mean % ID/g ± sd (*n* = 4). Initially, high uptake of the radiotracer was observed at 1 h pi in all tissues, potentially related with high radioactivity levels in the blood. However, high uptake was found in the pancreas and tumor as well. This situation changed at 4 h pi, with the radioactivity clearing from background, both via the hepatobiliary pathway and the kidneys and urinary tract. The background activity further declined at 24 h pi in all tissues, including the gastrointestinal tract and the pancreas. In contrast, tumor uptake remained practically unchanged between 1 h and 24 h pi.

## 3. Discussion

The overexpression of GRPR in a variety of cancer types provides opportunities for diagnosis and therapy by means of peptide-radiopharmaceuticals directed to GRPR-positive lesions [16,18,19]. Initial studies with BBN-like internalizing radioagonists have revealed promising candidates for translation in prostate cancer patients. However, clinical testing of therapeutic analogs with higher amounts of potent agonists administered to patients was associated with acute adverse effects elicited in the gastrointestinal system by GRPR-activation. This unfavorable result prompted a shift of paradigm in the field of prostate cancer radiopeptides from GRPR-agonists to GRPR-antagonists [13,18]. Progress in this direction was facilitated by studies in preceding decades on GRPR-antagonist motifs developed by peptide chemists and applied with considerable success as anti-tumor agents in GRPR-positive xenografts in mice [24,25,26,27]. Consequently, a great number of promising GRPR-radioantagonists was developed and thoroughly studied in prostate cancer models [18]. Promising candidates for clinical translation were selected from this pool of new agents, and showed excellent tumor targeting properties in prostate cancer patients [28,29,30,31,32].

Such analogs have been proposed for prostate cancer theragnostics, either alone or in combination with other modalities directed to alternative and/or complementary biomolecular targets, such as, for example, radiolabeled inhibitors of prostate-specific membrane antigen (PSMA) on cancer cells [33,34]. However, the invaluable preclinical and clinical experience hitherto acquired from recent developments in prostate cancer radiopharmaceuticals can be elegantly exploited for breast cancer theragnostics as well. This option is based on the high GRPR-expression documented in mammary carcinoma, especially in estrogen receptor-positive forms of the disease [10,14,16]. Notably, visualization of primary and metastatic breast cancer by some of these analogs was successfully demonstrated in a small number of patients employing PET/CT [28,35].

Following the above rationale, we decided to assess the visualization prospects of [^68^Ga]NeoBOMB1 in breast cancer, starting from the preclinical evaluation of the [^67^Ga]NeoBOMB1 surrogate in T-47D cells and subcutaneous xenografts in mice. Our decision was triggered by positive results from previous reports in PC-3 models that highlighted the theragnostic potential of [^67/68^Ga/^111^In/^177^Lu]NeoBOMB1 radioligands in prostate cancer and by the excellent visualization capacity of [^68^Ga]NeoBOMB1 in GRPR-positive lesions in prostate cancer patients [19,20]. As a suitable preclinical model for reliable evaluation of [^67^Ga]NeoBOMB1 in breast cancer we selected the T-47D cell line [22]. Previous studies have demonstrated superior expression of the GRPR in T-47D cells among a series of eight human breast cancer cell lines [23]. This finding was later confirmed by comparing the cell-uptake of a BBN-radioligand across nine breast cancer cell lines [14]. Although a direct correlation of GRPR-status and estrogen receptor expression could not be unequivocally demonstrated in the cell lines of this latest study, such a relationship could be established in biopsy specimens from breast cancer patients [14,16].

First, we were interested to test the binding affinity of both unlabeled NeoBOMB1 and its ^nat^Ga-metalated counterpart for the GRPR, in relation to previous findings from assays in PC-3 cell membranes [19]. In the present assay, however, we incubated live T-47D cells for 3 h at 4 °C to prevent inadvertent enzymatic proteolysis of tested peptides and/or the [^125^I-Tyr^4^]BBN radioligand during the assay; this period of time was deemed adequate for reaching equilibrium, on the basis of previous reports [23,36,37]. Interestingly, high and indistinguishable GRPR-affinity was exhibited by both NeoBOMB1 and [^nat^Ga]NeoBOMB1 in T-47D cells (Figure 2a), in agreement with results in PC-3 cells [19]. This affinity was found to be superior compared to the affinities reported for a series of unlabeled DOTA/NOTA-X-BBN(7–14) conjugates tested in T-47D cells in suspension (IC_50_ range: 5.9–78.5 nM; NOTA: 1,4,7-triazacyclononane-triacetic acid) [36,37]. After successful labeling of NeoBOMB1 with ^67^Ga, [^67^Ga]NeoBOMB1 rapidly bound to T-47D cells at 37 °C. The bulk of radioactivity remained at the cell-membrane, while only a small portion internalized in the cells, as expected for a radioantagonist. Cell-association was GRPR-specific at all time points tested, increasing with time to a maximum of 57.72 ± 0.66% of total added activity at 2 h incubation.

High receptor affinity and massive binding of peptide-radioligands to cells expressing their cognate receptor are not the only prerequisites for successful in vivo tumor targeting. The notorious propensity of peptides to proteolytic degradation, necessary for regulating their action, has become a serious hurdle in the development of peptide-based drugs, including peptide radiopharmaceuticals [2,38]. However, *N*-terminal capping by the metal-chelate will “protect” radiopeptides from aminopeptidases. Furthermore, strategic replacements of key-amino acids or modifications of the peptide-backbone per se may enhance the resistance of radiopeptides to peptidases, often at the cost of other important biological traits [38]. The metabolic stability of radiopeptides, typically determined by in vitro incubation in plasma or serum, may be overestimated by disregarding the action of peptidases anchored on epithelial cells of vessels and other tissues of the body [39]. Previous studies on BBN-like radiopeptides have unequivocally demonstrated the limitations of in vitro methods and implicated neutral endopeptidase (NEP) in their in vivo breakdown [40,41]. Since then, coinjection of NEP-inhibitors has been shown to improve bioavailability, and hence tumor uptake, of many radiopeptides [39]. Although radiolabeled GRPR-antagonists have been shown to be more resistant to the action of NEP, transient in vivo NEP-inhibition turned out to significantly improve their theragnostic potential, as well [14,42]. In the case of [^67^Ga]NeoBOMB1, however, an unprecedented high metabolic stability was documented in peripheral mouse blood (Figure 3), even at 30 min pi. This result confirmed in vivo previous reports on the high in vitro metabolic stability of the Ac-His-Trp-Ala-Val-Gly-His-NH-CH[CH_2_-CH(CH_3_)_2_]_2_ GRPR-antagonist motif [21].

It is interesting to observe how all of the above favorable in vitro and in vivo qualities of [^67^Ga]NeoBOMB1 translated into tumor-targeting capacity in experimental animal models. It should be noted that the development of subcutaneous T-47D tumors in SCID mice required treatment with estrogens [14]. In our model, estrogens were administered to mice via their drinking water supply a week prior to inoculation. Estrogen treatment continued over a period of about 8 weeks until well-palpable tumors formed. In a first set of experiments conducted at the 4 h pi time interval, we were interested to investigate the effect of peptide mass on the uptake of [^67^Ga]NeoBOMB1 in the T-47D xenografts and overall pharmacokinetics, by administering two distinct peptide doses of 10 pmol (specific activity: 3.7 MBq/nmol) and 200 pmol (specific activity: 0.185 MBq/nmol). Previous experiments in PC-3 tumor bearing mice revealed lower background radioactivity levels in combination with similar tumor uptake at the higher peptide dose, a phenomenon still under investigation [20]. In favor of theragnostic perspectives, there was a significant reduction of uptake in the GRPR-rich mouse pancreas for both [^68^Ga]NeoBOMB1 and [^177^Lu]NeoBOMB1 with rapidly increasing tumor-to-pancreas ratios for the 200 pmol dose [20]. Likewise, a very significant reduction of background radioactivity in the higher peptide dose was also observed for [^67^Ga]NeoBOMB1 in the present study, especially in the pancreas, while uptake in the T-47D tumors remained unaffected (Table 1). Biodistribution at this higher peptide dose was also studied at other time points (Table 2), revealing a significant and prolonged uptake of [^67^Ga]NeoBOMB1 in the T-47D implants from 1 to 24 h pi and a gradual clearance from background tissues, thus raising hopes for the applicability of [^177^Lu]NeoBOMB1 for breast cancer therapy. It should be noted that [^67^Ga]NeoBOMB1 displayed higher uptake and retention in the T-47D tumors in comparison to a series of ^64^Cu-labeled DOTA/NOTA-X-BBN(7–14) agonists in the same animal model (e.g., 8.67 ± 2.88% ID/g vs. <4% ID/g for the best ^64^Cu-radioligand in the series) [36,37]. It would be worth investigating to what extent the lower tumor targeting of these analogs is a result of a poorer metabolic stability in the mouse blood stream, or influenced by other factors. Interestingly, the uptake of another ^111^In-labeled GRPR-radioantagonist in T-47D tumors in mice did not exceed ≈5% ID/g at 200 pmol peptide dose at 4 h pi, even during in situ NEP-inhibition by coinjection of phosphoramidon. The latter intervention significantly improved the bioavailability and uptake of the ^111^In-radioligand in the T-47D implants [14].

To our knowledge, this is the first preclinical study of a GRPR-radioantagonist for PET fully conducted in breast cancer models. Results from this study, revealed the high GRPR-affinity, unprecedented in vivo stability and prolonged retention of [^67^Ga]NeoBOMB1 in GRPR-positiveT-47D tumor models. These findings are very promising for clinical translation in breast cancer patients applying [^68^Ga]NeoBOMB1 and PET/CT, as documented for other GRPR-radioantagonists [28,35]. Prolonged retention in the breast cancer model up to 24 h pi and substantial reduction of background radioactivity by tuning of peptide-dose, raise significant hopes for theragnostic application of the [^68^Ga/^177^Lu]NeoBOMB1 pair in breast cancer. This perspective will be explored following the precedent path paved by the same theragnostic pair in prostate cancer, currently under extensive investigation.

## 4. Materials and Methods

### 4.1. Peptides and Radioligands

NeoBOMB1 (DOTA-*p*-aminomethylaniline-diglycolic acid-DPhe-Gln-Trp-Ala-Val-Gly-His-NH-CH[CH_2_-CH(CH_3_)_2_]_2_; Figure 1) was purchased from PiChem (Graz, Austria), while [Tyr^4^]BBN (Tyr^4^-bombesin, Pyr-Gln-Arg-Tyr-Gly-Asn-Gln-Trp-Ala-Val-Gly-His-Leu-Met-NH_2_) was obtained by PSL GmbH (Heidelberg, Germany).

#### 4.1.1. Preparation and Quality Control of [^67^Ga]NeoBOMB1

Lyophilized NeoBOMB1 was dissolved in HPLC-grade H_2_O (2 mg/mL) and 50 μL aliquots thereof were stored in Eppendorf Protein LoBind tubes at −20 °C. For labeling, ^67^GaCl_3_ in dilute HC1 at an activity concentration of 18.4–27.5 GBq/mL was used (IDB Holland B.V.s., Baarle-Nassau, The Netherlands). [^67^Ga]NeoBOMB1 was obtained at specific activities of 3.7–7.4 MBq ^67^Ga/nmol NeoBOMB1. Briefly, 3–15 nmol of NeoBOMB1 was mixed with 50–150 μL of 1 M pH 4.0 sodium acetate buffer and 5–15 μL of ^67^GaCl_3_ (11–111 MBq). The mixture was incubated at 90 °C for 30 min, and Na_2_-EDTA (0.1 M, pH 4.0) was added to a final concentration of 1 mM [19].

Reversed-phase HPLC was performed on a Waters Chromatograph (Vienna, Austria) based on a 600E multisolvent delivery system coupled to a Waters 2998 (Vienna, Austria) photodiode array detector and a Gabi gamma-detector (Raytest, RSM Analytische Instrumente GmbH, Straubenhardt, Germany). Data processing and chromatography were controlled by the Empower Software (Vienna, Austria) by Waters (Vienna, Austria). For quality control of the labelling solution a Symmetry Shield RP18 cartridge column (5 μm, 3.9 mm × 20 mm, Waters) was eluted with the following linear gradient: 100% A and 0% B to 0% A and 100% B in 50 min (A = 0.1% aqueous TFA (*v*/*v*) and B = MeCN). Samples of [^67^Ga]NeoBOMB1 were analyzed before and after completion of all biological experiments, *t*_R_([^67^Ga]NeoBOMB1) = 20.0 min (Figure 1b).

#### 4.1.2. Preparation of [^125^I-Tyr^4^]BBN

[Tyr^4^]BBN and ^125^I (MDS Nordion, Vancouver, BC, Canada) were used for the preparation of [^125^I-Tyr^4^]BBN. Radioiodination was performed by the chloramine-T methodology, as previously described [43,44]. The forming sulfoxide (Met^14^=O) was reduced by dithiothreitol and [^125^I-Tyr^4^]BBN was isolated in non-carrier added form by HPLC. Methionine was added to the purified radioligand solution to prevent re-oxidation of Met^14^ to the corresponding sulfoxide and the resulting stock solution in 0.1% BSA-PBS was kept at −20 °C; aliquots thereof were used for competition binding assays (specific activity of 2.2 Ci/μmol). Samples were measured for radioactivity in an automatic well-type gamma counter (NaI(Tl)] crystal, Canberra Packard Auto-Gamma 5000 series instrument, Schwadorf, Austria).

#### 4.1.3. Preparation of [^nat^Ga]NeoBOMB1

NeoBOMB1 (600 μg) was incubated with a threefold molar excess of ^nat^Ga(NO_3_)_3_·9H_2_O (Alfa-Ventron, Ward Hill, MA, USA) in acetate buffer (pH 4) at 90 °C for 30 min. The excess ^nat^Ga was then scavenged by addition of EDTA. Complete ^nat^Ga-metalation of NeoBOMB1 was verified by analytical HPLC, using an XTerra RP18 (5 μm, 3.9 mm × 20 mm, Waters) cartridge column [19]. The column was eluted at 1 mL/min flow rate with the following linear gradient: 100% A and 0% B to 30% A and 30% B in 30 min; (A = 0.1% aqueous TFA (*v*/*v*) and B = MeCN); *t*_R_(NeoBOMB1) = 26.5 min and *t*_R_([^nat^Ga]NeoBOMB1) = 27.9 min.

### 4.2. In Vitro Assays

#### 4.2.1. Cell Lines and Culture

Human ductal breast carcinoma T-47D cells endogenously expressing the GRPR were used in all biological assays [14,22,23]. Cells were cultured in RPMI supplemented with 10% FBS, 100 U/mL penicillin and 100 µg/mL streptomycin, and kept in a controlled humidified atmosphere containing 5% CO_2_ at 37 °C. Passages were performed weekly using a trypsin/EDTA (0.05%/0.02% *w*/*v*) solution.

#### 4.2.2. Competition Binding Assay in T-47D Cells

Competition binding experiments against [^125^I-Tyr^4^]BBN were performed with NeoBOMB1, [^nat^Ga]NeoBOMB1, or [Tyr^4^]-BBN (reference) in T-47D cells. Cells were seeded in 12-well plates 24 h in advance (0.6 × 10^6^ cells per well). On the day of the experiment, cells were placed on ice and washed with chilled washing medium (2 × 1 mL, RPMI supplemented with 1% heat inactivated FBS). The following solutions were then added in each well: binding medium (400 μL, 50 mM HEPES pH 7.4, 1% BSA, 5.5 mM MgCl_2_, 10 mM bacitracin), binding buffer (60 μL) containing increasing concentration of test peptide and radioligand solution in binding buffer (140 μL, ≈40,000 cpm per well). Cell-triplicates of each concentration point were incubated for 3 h at 4 °C (to rule out degradation of ligand and/or radioligand). Incubation was terminated by removing the supernatants with aspiration. Cells were washed with chilled washing buffer (1 × 1 mL), and 1 N NaOH was added to lyse the cells (2 × 0.6 mL). Lysates were collected, combined and counted for their radioactivity content in a γ-counter. The IC_50_ values were calculated adopting nonlinear regression according to a one-site model applying the PRISM^TM^ 2 program (GraphPad Software, San Diego, CA, USA). Nonspecific binding was defined as the amount of cell-bound activity in the presence of 1 μM [Tyr^4^]BBN. Results represent the mean values ± sd of three independent experiments performed in triplicate.

#### 4.2.3. Internalization Assay in T-47D Cells

The overall cell association—internalization of [^67^Ga]NeoBOMB1 was assessed in T-47D cells. Briefly, T-47D cells were seeded in six-well plates (~1 × 10^6^ cells per well) 24–48 h before the experiment. Approximately 50,000 cpm of [^67^Ga]NeoBOMB1 (corresponding to 250 fmol total peptide in 150 μL of 0.5% BSA/PBS) was added alone (total) or in the presence of 1 μM [Tyr^4^]BBN (non-specific). Cells were incubated at 37 °C for 15 min, 30 min, 1 h and 2 h intervals; incubation was interrupted by placing the plates on ice, removing the supernatants and rapid rinsing with ice-cold 0.5% BSA/PBS. Cells were then treated 2 × 5 min with acid wash buffer (2 × 0.6 mL, 50 mM glycine buffer pH 2.8, 0.1 M NaCl) at room temperature and supernatants were collected (membrane-bound fraction). After rinsing with chilled 0.5% BSA/PBS, cells were lyzed by treatment with 1 N NaOH (2 × 0.6 mL) and lysates were collected (internalized fraction). Sample radioactivity was measured in the γ-counter and total cell-associated (internalized + membrane bound) radioactivity was determined vs. total added activity. Results represent the average values ± sd of three experiments performed in triplicate.

### 4.3. Animal Studies

#### 4.3.1. In Vivo Stability Tests

[^67^Ga]NeoBOMB1 was injected as a 100 μL bolus (11–22 MBq, 3 nmol total peptide) in the tail vein of male Swiss albino mice (30 ± 5 g, NCSR “Demokritos” Animal House Facility). Mice were anesthetized, and blood (0.5–1 mL) was collected from the heart at 5 and 30 min post injection (pi). Animals were euthanized and blood directly drawn from the heart was transferred in a pre-chilled EDTA-containing Eppendorf tube on ice. Blood samples were centrifuged for 10 min at 2000× *g* at 4 °C and plasma was collected. After addition of an equal volume of ice-cold MeCN the mixture was centrifuged for 10 min at 15,000× *g* at 4 °C. The supernatant was concentrated under a N_2_-flux at 40 °C to 0.05–0.1 mL, diluted with saline (0.4 mL), filtered through a 0.22 μm Millex GV filter (Millipore, Milford, MA, USA) and analyzed by RP-HPLC. The Symmetry Shield RP18 (5 μm, 3.9 mm × 20 mm) column was eluted at a flow rate of 1.0 mL/min with the following linear gradient: 100% A and 0% B to 50% A and 50% B in 50 min; (A = 0.1% aqueous TFA (*v*/*v*) and B = MeCN). The *t*_R_ of the intact radiopeptide was determined by coinjection with the [^67^Ga]NeoBOMB1 reference in the HPLC.

#### 4.3.2. Induction of T-47D Xenografts in SCID Mice

Female SCID mice (15 ± 3 g, six weeks of age animals at the day of arrival, NCSR “Demokritos” Animal House Facility) were acclimatized under aseptic conditions and treated with estrogens (receiving 4 mg/L β-estradiol, Sigma-Aldrich, St. Louis, MO, USA,, in their drinking water,) for a week prior to inoculation. A ≈ 150 μL bolus containing a suspension of ≈1.2 × 10^7^ freshly harvested human T-47D cells suspended in Matrigel (Corning Life Sciences, Inc., Bedford, MA, USA) was subcutaneously injected in their flanks. The animals were kept under estrogen treatment for additional 8 weeks till they developed well-palpable tumors (100–200 mg) at the inoculation sites.

#### 4.3.3. Biodistribution in T-47D Xenograft-Bearing SCID Mice

On the day of the experiment animals were allowed access to water, but not to food. Animals in 3 groups of 4 received a 100 μL bolus of [^67^Ga]NeoBOMB1 (37 kBq, corresponding to 10 pmol, or 200 pmol total peptide or 40 nmol for in vivo GRPR-blockade for each of the 3 groups) was injected in the tail vein; animals were euthanized at 4 h pi and dissected. Samples of blood, tumors and organs of interest were collected, weighed and measured for radioactivity in the gamma counter. Intestines and stomach were not emptied of their contents. In another experimental setting, mice in 3 groups of 4 received a 100 μL bolus of [^67^Ga]NeoBOMB1 (37 kBq, 200 pmol); they were euthanized at 1 h, 4 h and 24 h pi and biodistribution was conducted as described above. Statistical analysis was conducted using the unpaired two-tailed Student’s *t*-test; values *p* < 0.05 were considered statistically significant.

All animal experiments were carried out in compliance with European and national regulations and after approval of protocols by national Authorities (protocol #6461approved by Prefecture of Athens - in #EL 25 BIO 021 certified facility).

## 5. Conclusions

The present preclinical study has revealed favorably prolonged retention for [^67^Ga]NeoBOMB1 in experimental T-47D breast tumors in mice as well as significant reduction of background radioactivity by tuning peptide-dose. These qualities are of great promise for theragnostic application of the respective [^68^Ga/^177^Lu]NeoBOMB1 pair in breast cancer management. This option is currently being actively investigated.

## Figures and Tables

**Table 1:** [^67^Ga]NeoBOMB1 biodistribution data, as % ID/g mean ± sd, *n* = 4; at 4 h pi in T-47D Xenograft-Bearing SCID Mice; results in 3 different NeoBOMB1 doses are included in each column, with 40 nmol administered for in vivo GRPR-blockade.

Tissue/Dose	10 pmol ^1^	200 pmol ^2^	40 nmol ^3^
Blood	0.53 ± 0.04	0.42 ± 0.06	0.46 ± 0.03
Liver	3.37 ± 0.19	6.50 ± 0.34	9.67 ± 0.75
Heart	0.26 ± 0.01	0.34 ± 0.04	0.38 ± 0.02
Kidneys	3.40 ± 0.45	2.76 ± 0.26	3.95 ± 0.94
Stomach	4.36 ± 0.56	2.38 ± 0.13	0.42 ± 0.10
Intestines	16.71 ± 2.18	15.13 ± 1.52	11.08 ± 2.71
Spleen	1.02 ± 0.09	0.70 ± 0.09	0.79 ± 0.11
Muscle	0.10 ± 0.03	0.12 ± 0.01	0.16 ± 0.06
Lungs	0.50 ± 0.06	0.43 ± 0.06	0.42 ± 0.02
Femur	0.58 ± 0.06	0.28 ± 0.01	0.31 ± 0.02
Pancreas	206.29 ± 17.35	42.46 ± 1.31	1.17 ± 0.11
Tumor	9.52 ± 2.15	7.79 ± 1.54	0.64 ± 0.10

^1^ Corresponding to animal groups injected with a specific activity of 3.7 MBq/nmol; or ^2^ 0.185 MBq/nmol; ^3^ in vivo GRPR-blockade mice group.

**Table 2:** [^67^Ga]NeoBOMB1 biodistribution data, as % ID/g mean ± sd, *n* = 4; at 1, 4 and 24 h pi in T-47D Xenograft-Bearing SCID Mice at a 200 pmol peptide dose ^1^.

Tissue/Time	1 h	4 h	24 h
Blood	5.78 ± 0.29	0.52 ± 0.03	0.15 ± 0.01
Liver	25.66 ± 1.97	7.05 ± 0.31	2.26 ± 0.18
Heart	1.94 ± 0.09	0.52 ± 0.04	0.22 ± 0.04
Kidneys	7.33 ± 0.47	3.10 ± 0.75	2.15 ± 0.42
Stomach	5.89 ± 1.02	2.66 ± 0.16	2.07 ± 0.43
Intestines	10.62 ± 0.87	18.52 ± 2.12	2.31 ± 0.34
Spleen	1.71 ± 0.50	0.89 ± 0.12	0.46 ± 0.08
Muscle	0.58 ± 0.11	0.17 ± 0.00	0.13 ± 0.01
Lungs	2.88 ± 0.20	0.47 ± 0.04	0.15 ± 0.02
Femur	0.73 ± 0.11	0.37 ± 0.02	0.24 ± 0.08
Pancreas	32.45 ± 0.78	36.86 ± 3.58	21.33 ± 2.77
Tumor	7.40 ± 0.68	8.67 ± 2.88	7.89 ± 1.13

^1^ All animal groups were injected with a specific activity of 0.185 MBq/nmol.
